# Spontaneous remission of eosinophilic granuloma of the maxilla after incisional biopsy: a case report

**DOI:** 10.1186/s13005-016-0118-9

**Published:** 2016-06-02

**Authors:** Alex Vargas, Hernán Ramírez, Pablo Ramírez, Camila Foncea, Bernardo Venegas, Paula Astorga

**Affiliations:** DDS. Associated Professor of the Oncology and Maxillofacial Surgery Department, Medicine Faculty, Pontificia Universidad Católica de Chile, Santiago, Chile; Assistant Professor of Hematology Department, Medicine Faculty, Pontificia Universidad Católica de Chile, Santiago, Chile; Resident of Oral and Maxillofacial Surgery Program, Medicine Faculty, Pontificia Universidad Católica de Chile, Santiago, Chile; Oral Pathologist at the University of Talca and Carlos Van Buren Hospital of Valparaíso, Valparaíso, Chile; Observer of the Oncology and Maxillofacial Surgery Department, Medicine Faculty, Pontificia Universidad Católica de Chile, Santiago, Chile

**Keywords:** Langerhans cell histiocytosis, Eosinophilic Granuloma, Maxillary bone

## Abstract

**Background:**

Langerhans cell histiocytosis (LCH), previously known as Histiocytosis X, is an infrequent disease that congregates a wide spectrum of clinical presentations with variable systemic involvement. Unification of these diseases under only one category is based on the almost identical histopathologic features of the lesions, but the etiology and proper approach for each presentation remains controversial. The localized alternative of Langerhans cell histiocytosis (LLCH), known as Eosinophilic Granuloma (EG) of bone, is the predominant clinical presentation of LCH. The maxilla is involved in 1 % of the head and neck region cases, representing an uncommon condition in this area.

**Case Presentation:**

In this clinical case report, it is described a case of a 16-year-old male patient with an asymptomatic osteolytic lesion at first upper left molar apical level, a finding detected on control radiographic images was reported as “Monostotic Eosinophilic granuloma of the maxillary bone”, which was later confirmed through an incisional biopsy. A surgical excision was initially planned, but finally it was not performed due to a spontaneous healing of the lesion after the incisional biopsy.

**Conclusions:**

The presented case supports a conservative approach in the management of solitary EG of maxillary and mandibular bone lesions and even supports an expectant attitude in the course of treatment given the possibility of a spontaneous regression after the biopsy, especially in small lesions.

## Background

Lichtenstein and Jaffe introduced the term Histiocytosis X in 1953 to encompass a group of uncommon granulomatous proliferative disorders characterized by the presence of histiocytic cells [1, 2]. The Eosinophilic granuloma is traditionally considered as one of the three clinical manifestations of Histiocytosis X, which also includes Hand-Schuller-Christian and Letterer-Siwe diseases [3].

Later in 1985 the generic name of this diseases was changed to Langerhans Cell Histiocytosis (LCH), in recognition to the principal component of the proliferative cell population [2]. As LCH is an uncommon disease, only a limited number of large surveys or randomized clinical trials are available in scientific literature and many treatment details remain either obscure or controversial [4, 5].

Only 1 % of the head and neck region cases of EG involves the maxillary bone [1]. For this reason, the purpose of this case report is to describe a rare case of Eosinophilic Granuloma in a 16-year-old patient, which healed spontaneously after incisional biopsy, and to present a brief review of the available literature on the topic.

## Case presentation

A 16-year-old male patient, without any relevant aspects in his medical history except a Non-Hodgkin lymphoma of his father, which is currently being treated, and a breast cancer of his mother, also in treatment. The patient was derived from the Hematology Unit for evaluation and definitive treatment of an osteolytic lesion in the left maxillary region with an evolution of nearly 2 months. Thirty days before, the patient had consulted at a dental service for a routine dental exam in which a radiolucent image appeared in the radiographic study. The patient underwent a biopsy of an asymptomatic osteolytic lesion located at apical level of the first upper left molar.

In his admission to our service, the oral and maxillofacial clinical examination did not show facial asymmetry, swelling or solution of continuity in the left maxillary alveolar ridge. The patient only had a slight and generalized gingival inflammation, and no other findings.

**Fig. 1:**
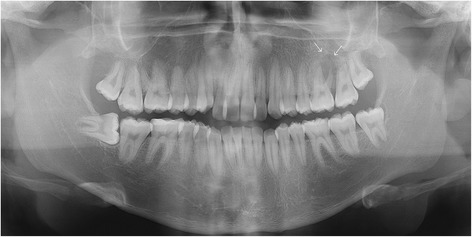
Panoramic radiograph obtained before biopsy, showing an osteolytic lesion in relation to periapex of the first upper left molar and projecting toward the floor of the maxillary sinus (white arrows)

**Fig. 2:**
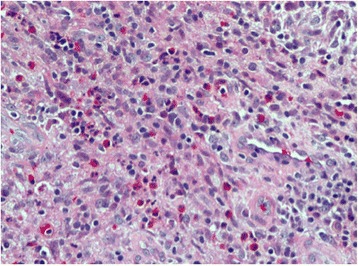
Histological analysis shows fibrous connective tissue with lymphoplasmacytic inflammatory infiltrate and strong presence of eosinophils (Hematoxylin-Eosin. 40×)

**Fig. 3:**
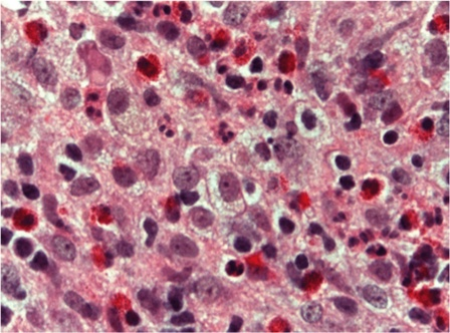
Histiocyte-like cells with clear cytoplasm and pale rounded or some indented nuclei can be observed. Prominent eosinophilic granules and also some neutrophils are present (Hematoxylin-Eosin. 100×)

He brought images of a recent dental study (panoramic radiography) and a report of a biopsy performed 30 days before. The imaging study reported an osteolytic lesion in the left maxillary ridge, extending between the first and second upper left molars, with projection to the maxillary sinus [Fig. 1]. The biopsy report concluded that the sample was consistent with bone monostotic Eosinophilic granuloma (Langerhans histiocytosis). The histophatological findings of the performed biopsy, compatible with Eosinophilic granuloma, are showed in the Fig. 2 and Fig. 3.

To complete the case study, additionally to the routine preoperative laboratory tests (blood count, biochemical profile and coagulation tests), a cone-beam CT (CBCT) was requested, and to rule out compromise of other bones, a scintigraphy was requested. Previously performed paraffin embedded samples of biopsy were also requested.

**Fig. 4:**
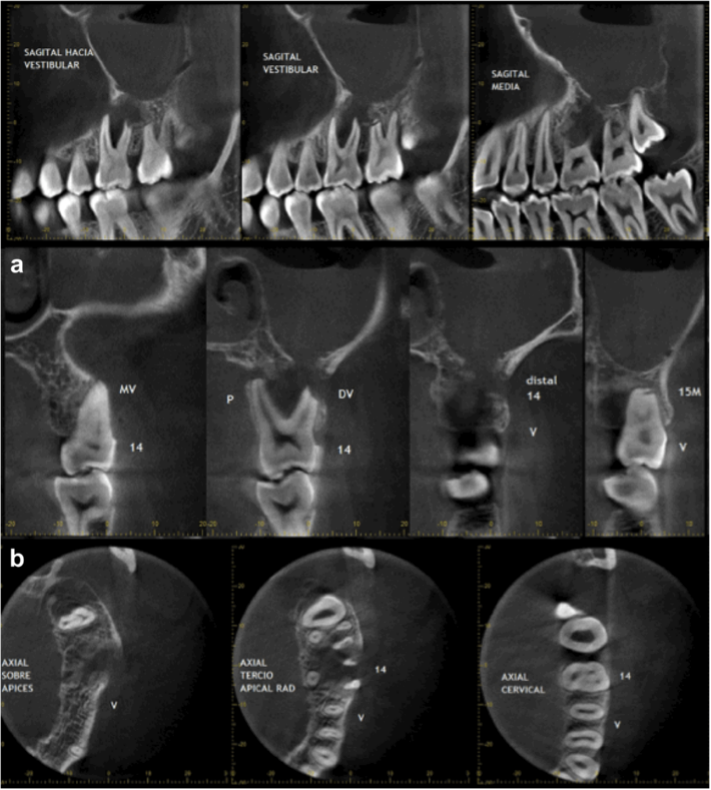
The CBCT showed veiling of the left maxillary sinus, soil discontinuity of ipsilateral sinus, related with the osteolytic lesion around the roots of the upper left first molar; and extensive loss of continuity of the buccal bone plate, compatible with post-surgical biopsy changes

**Fig. 5:**
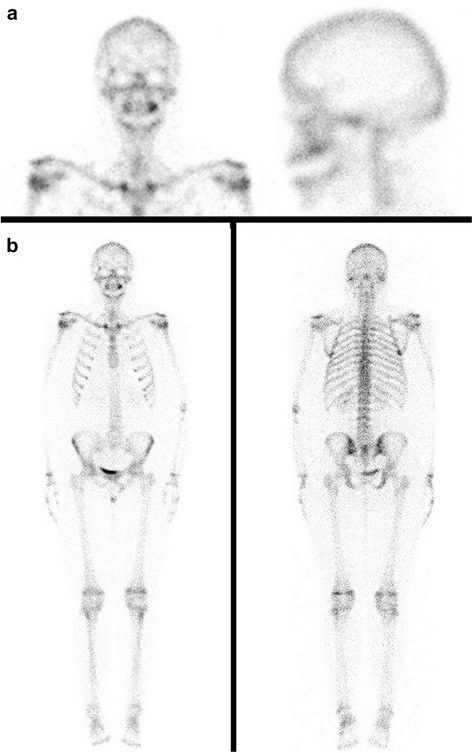
Bone scintigraphy showed an increase in osteoblast activity in the left maxillary bone, related with the known bone injury. No other findings of pathological significance in the rest of the study

Laboratory tests were normal. The new CBCT [Fig. 4] showed inflammatory changes in relation to the left maxillary sinus with great opacification, an area of osteolysis in connection with the roots of the upper left first molar, causing soil discontinuity of the ipsilateral sinus; and extensive loss of continuity of the oral bone plate, compatible with post-surgical biopsy changes. Bone scintigraphy showed an increase in osteoblast activity in the left maxillary bone, related with the known bone injury. There were no other findings of pathological significance in the rest of the study [Fig. 5].

**Fig. 6:**
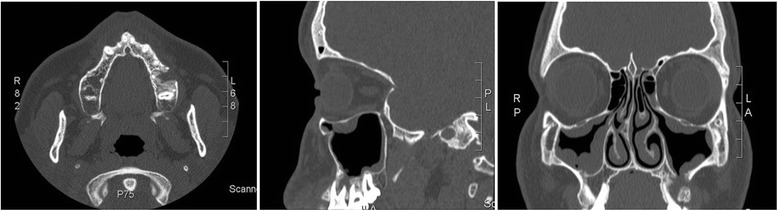
CT images of sinus commitment, after the biopsy and before complete surgical removal of the lesion

Given these images of sinus compromise, nasal congestion and subsequent discharge reported by the patient, it was decided to complete the study with computed tomography (CT) of the sinuses before the complete surgical removal of the lesion [Fig. 6], and refer the patient to the assessment and care of an otolaryngologist.

**Fig. 7:**
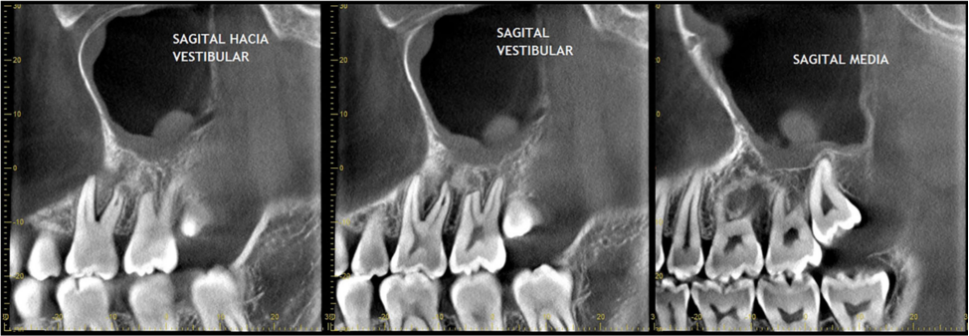
CBCT images of three months after first examination, showing recovery of the left maxillary sinus transparency although the mucosal thickening of the floor persists

The patient came back to us 3 months after his first examination to schedule his surgical procedure, which prompted new imaging study to redefine treatment [Fig. 7].

**Fig. 8:**
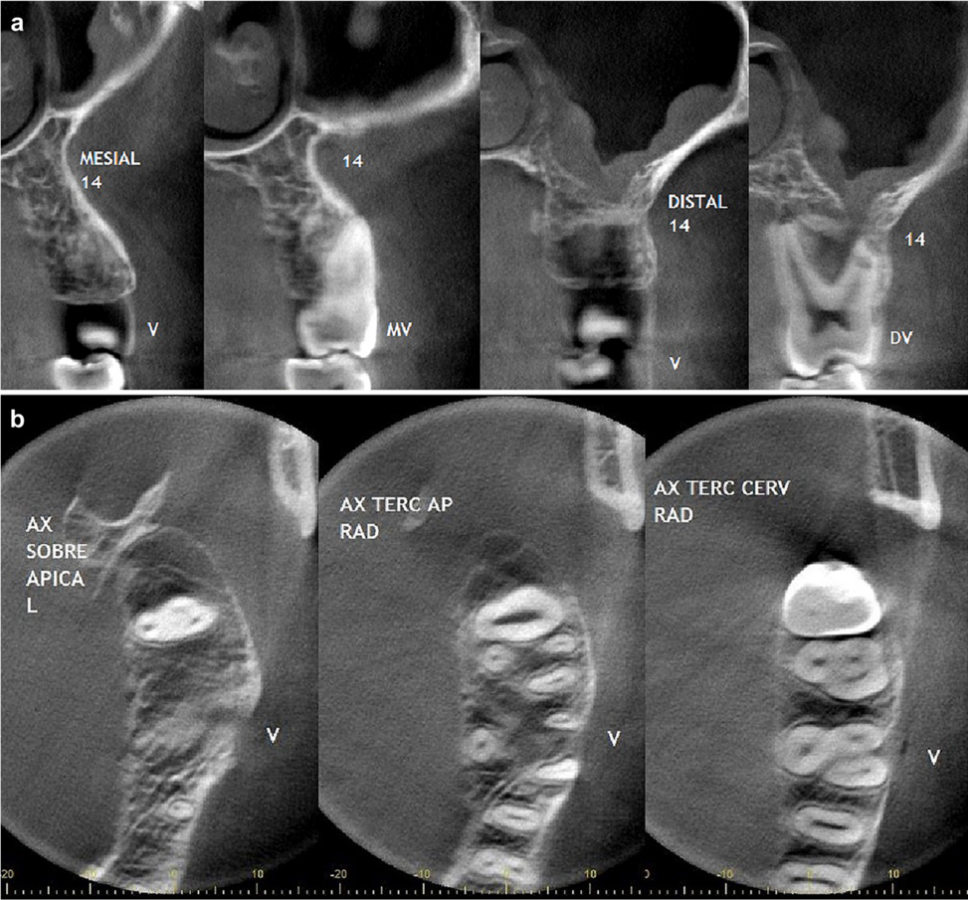
Remarkable is the presence of new bone newly formed in the periphery and between the roots compromised by the initial injury and the decrease in density areas found in previous pictures

Later studies showed recovery of the left maxillary sinus transparency in spite of the persistence of mucosal thickening of the sinus floor. Newly formed bone in the periphery and between the roots was compromised by the initial injury, and a decrease of the hypodense areas found in the previous examination was also observed [Fig. 8].

**Fig. 9:**
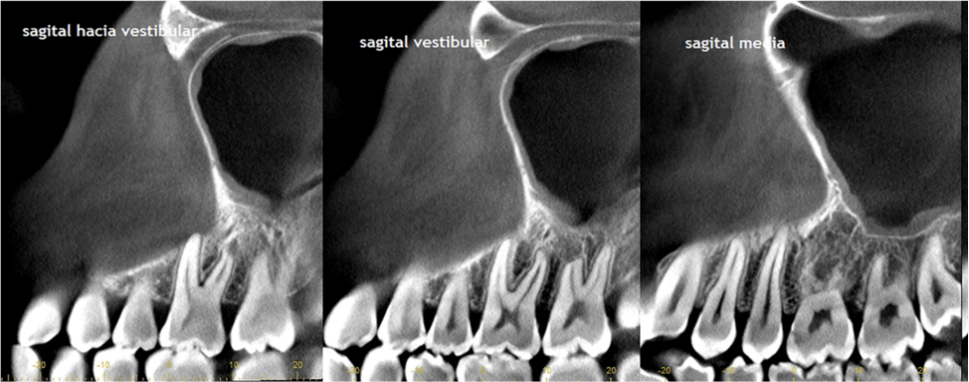
CT images show the grade of bone repair in the originally affected area, nine months after biopsy

**Fig. 10:**
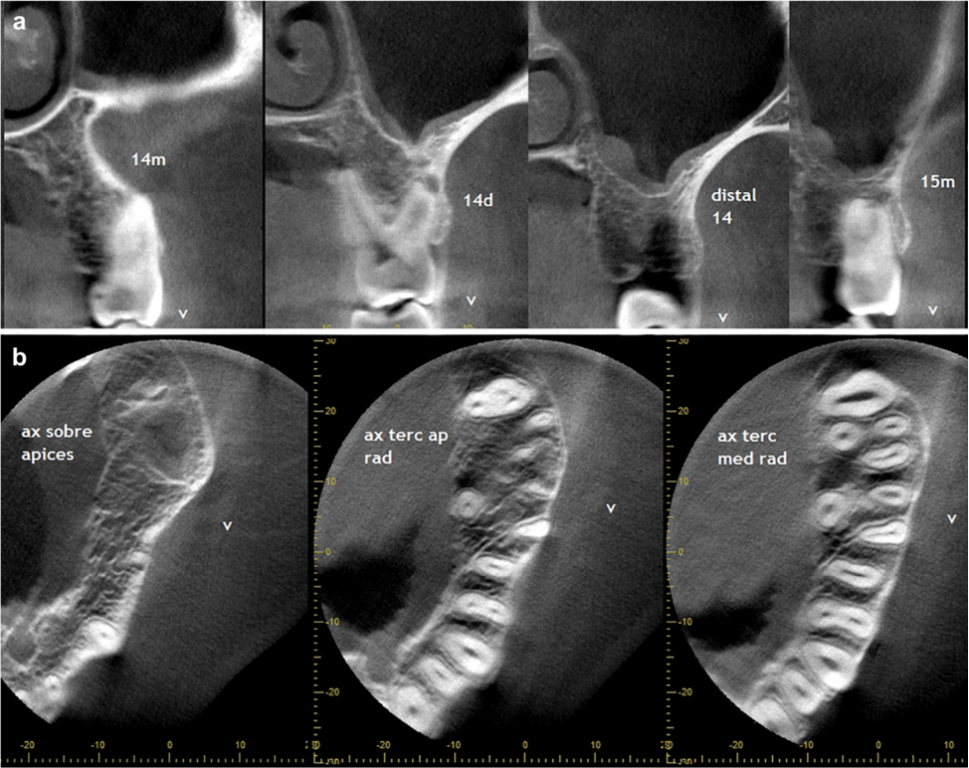
Nine months after the biopsy was performed, it is possible to see the repaired bone in the area initially affected

These findings were consistent with spontaneous remission of the osteolytic lesion after the biopsy. The patient underwent regular checks to ensure no recurrence of his original injury; the last check was 9 months after the initial biopsy. Figures 9 and 10 show the grade of bone repair in the originally affected area. Since the EG is a benign lesion, which developed a spontaneous remission in this case, our patient’s prognosis is excellent, as long he follows his regular checks in order to detect any early recurrence or new outbreaks for at least 5 years.

## Discussion

Langerhans cell histiocytosis is a group of disorders characterized for a clonal proliferation of Langerhans cells [2, 6], with a wide spectrum of clinical presentations, including solitary bone lesions with excellent prognosis, known as eosinophilic granuloma, and other systemic presentations with a multi-organic, disseminated and progressive course, previously known as Hand-Schüller-Christian syndrome and Letterer-Siwe disease [3]. The current classification differentiates between single system disease with one organ or system involved and multisystem disease with two or more systems involved that may include ‘risk organs’ (hematopoietic system, spleen and/or liver) [5].

This grouping of different entities has been based on the similarities of the histopathologic appearance of the histiocytic and eosinophilic proliferation and by the involvement of Langerhans cells precursors [7]. A presumptive diagnosis is made when the typical morphological features of Langerhans cells are found, and it is confirmed if it stains for S-100 protein, CD1a and/or CD207 (Langerin) antigens are positive [4]. The detection of cytoplasmic inclusion bodies known as Birbeck-Breatnach granules is another typical characteristic of LCH [8]. When the diagnosis is made, bone scan and PET are indicated to evaluate multiple involvements and to discard the possibility of polyostotic disease [9].

The cause and pathogenesis of these disorders remains unknown. It has been proposed that a basic immune defect may lead to proliferation of Langerhans cells; or that the Langerhans cell itself may carry a genetic defect leading to abnormal cellular proliferation [8]. A key feature of this neoplasm is its clonal derivation from a single cell, which does not necessarily mean malignancy [6]. On the other hand, it is reported that proinflammatory cytokines and chemokines as well as oncogenic B-type Raf kinase (BRAF) gene mutations are involved in the pathogenesis of EG [10]. BRAF mutations are present in several malignant neoplasias including papillary thyrd carcinoma [11]. Its finding in EG suggests a neoplastic condition of the lesion, concept that must be reevaluated considering the existence of spontaneous resolution of this disorder. Differentiation disorders of Langerhans cells have also been studied [12]. Recently, it has been proposed a model of pathogenesis of Langerhans cell Histiocytosis in which there is an initial inflammatory condition potentiated by Interleukin 1, continued by mutations of BRAF genes. Both processes are also being considered as potential therapeutic targets [13].

Also, HCL has been associated with other malignancies such as leukemias, lymphomas and other solid tumors. This may precede, occur concurrently or follow the diagnosis of LCH and should be considered at every clinical visit [1, 4]. Acute lymphoblastic leukemia and lymphoma occur more often prior to the diagnosis of LCH, but they may be diagnosed up to 5 years after LCH [4].

In our case, the patient also has a family history of malignancies, although there is no clear family association for the development of these disorders, we must consider this precedence and assure clinical evaluations over time.

Clinical course and prognosis of the disease depend on the age of initial manifestation as well as the location and the organs involved [8]. The anatomic site itself (particularly in bone involvement), the volume of involved soft tissue and local destruction degree have a greater impact on the outcome of the disease than the actual number of bones involved [5]. Multisystem disease (MS-LCH) generally has a worse prognosis and a more complex treatment. Fortunately, EG is the predominant clinical form of LCH and accounts for 50 to 60 % of all cases of LCH [14]. Any organ or system of the human body can be affected, but those more frequently involved are the skeleton (80 % of cases) and the skin (33 %) [4].

EG of bone is a disease with an incidence of one new case per 350,000 to 2 million per year [7, 8]. It is most common in teenagers and adults, with approximately 75 % of all patients younger than 20 years old [1, 8, 14] and 90 % under 40, with an average age of 19 [1]. Males are affected twice as often as women [1]. In the head and neck bone region, the compromised sites are skull (27 – 43 %), mandible (7 – 9 %), maxillary bone (1 %) and cervical vertebra (2 %), accordingly to the reported by Hicks J. et al., 2005 [1]. Lesions are unifocal in 50 – 75 % of the cases, being 3 times more common than multifocal involvement [1]. Our case report presents an interesting clinical case of a unifocal lesion of alveolar ridge of the maxillary bone, given its low incidence but especially for its spontaneous regression. Although literature reports spontaneous remission of EG in several locations including mandible, femur, intracranial bones, lungs and orbit bones [15–17]; no reference to a specific quantification of incidence was found. Several articles recommend considering the “wait and see” policy when this lesion is confirmed, but a closer and longer follow up appears to be strictly necessary.

The clinical spectrum of EG in maxillary and mandibular bones is quite varied and may mimic a variety of conditions, such as apical cysts, odontogenic tumors (e.g., ameloblastoma), non-odontogenic tumors (e.g., central giant cell granuloma), inflammatory diseases (e.g., osteomyelitis), vascular malformations, and malignancies [7, 14].

When there are clinical manifestations, the most common signs and symptoms in the oral cavity are intraoral mass, pain, gingival inflammation, bleeding, mobile teeth, oral mucosal ulcer, impaired healing, and halitosis [1, 7], and it is usually misdiagnosed as marginal periodontal infections [8]. On the other hand, lesion may occasionally remain asymptomatic, leading to underdiagnosis [9]. In our case the patient only referred localized swelling associated with gingival inflammation, with neither pain nor tooth mobility, and the lesion was detected with a routine panoramic radiography.

Due to incomplete understanding of the pathogenesis of LCH, treatment has been based on different empirical strategies, considering the extent of the disease and the severity at onset [4, 6]. There is also an agreement on the fact that single system disease has good prognosis with a high rate of spontaneous remission and negligible mortality [18]. With unifocal bone disease, complete excision of bone lesions (curettage) may be indicated if the lesion is small (<2 cm) and for lesions with a diameter of 2 to 5 cm, a biopsy and curettage is an option [4, 19]. In the present case, surgical excision was proposed initially, but apparently simple curettage during the diagnostic biopsy turned out in remission at the control with CT scan. Consequently, it was decided to keep an expectant attitude, maintaining controls until the lesion’s total remission. This option is the most conservative and less invasive, and it is consistent with other cases presented previously, which support a conservative approach concerning treatment [2, 20, 21].

Another approach to solitary lesions treatment is an intra lesional injection of steroids [22–24], however, the standard treatment for LCH is not yet clear and surgery is still the treatment of choice in these cases [8, 14]. Other therapeutic combinations of LCH include surgery, chemotherapy and radiation, either individually or together, which may be necessary in multisystem disease. New therapeutic strategies are represented by monoclonal CD-1a-antibody-therapy and gene transfer into hemopoietic progenitor cells [8] and gene therapy [6].

Excision with or without radiation therapy has led to more than 95 % disease-free survival. However, relapse occurs in about 10 % of cases. Surgery alone has a 12 % recurrence rate, compared with 25 % for radiation therapy alone and 19 % for both combined therapies [1].

Patients without a histologically confirmed diagnosis need to be carefully monitored by appropriate imaging for at least 6 months after diagnosis in order to reassess the need for biopsy and its justification, in order to exclude a malignancy [4]. In the present case, the patient has more than 1 year of postoperative controls. Besides, bone scintigraphy fortunately discards the possibility of polyostotic disease, which allowed us to maintain an expectant approach of the lesion’s regression, without the need of new surgery.

All patients should be followed for a sufficient time period, defined as (i) at least 5 years after the end of therapy; or (ii) 5 years after the last disease reactivation, in those who did not receive systemic therapy; or (iii) until final growth and pubertal development have occurred [4, 25]. In our case, since the pubertal growth of the patient is probably finished, a minimum of 5 years follow-up is necessary, to exclude recurrence.

## Conclusions

Clinicians who treat maxillofacial disorders should be aware of the possibilities of LCH diagnosis and subsequent spectrum of clinical presentations. Faced with a diagnosis of HCL is mandatory to carry out a thorough examination and discard their participation in other systems.

The main lesson of this case report is the usefulness and validity of the use of conservative approaches in the management of EG solitary maxillary bone lesions and that it even supports an expectant attitude toward the possibility of spontaneous regression only performing biopsy, especially in small lesions.

## Abbreviations

CBCT, cone beam CT; CT, computed tomography; EG, cosinophilic Granuloma; LCH, lzangerhans cell histiocytosis; LLCH, localized langerhans cell histiocytosis; MS-LCH, lultisystem langerhans cell histiocytosis.
